# Sensitization to Cockroach Allergen: Immune Regulation and Genetic Determinants

**DOI:** 10.1155/2012/563760

**Published:** 2012-01-09

**Authors:** Peisong Gao

**Affiliations:** Department of Medicine, Johns Hopkins Asthma and Allergy Center, Johns Hopkins University School of Medicine, Baltimore, MD 21224, USA

## Abstract

Asthma is a major public health concern. Cockroach allergen exposure and cockroach allergic sensitization could contribute to the higher prevalence of asthma. However, the underlying immune mechanism and the genetic etiology remain unclear. Recent advances have demonstrated that several receptors (PAR-2, TLRs, CLRs) and their pathways mediate antigen uptake from the environment and induce allergies by signaling T cells to activate an inappropriate immune response. Cockroach-derived protease can disturb airway epithelial integrity via PAR-2 and leads to an increased penetration of cockroach allergen, resulting in activation of innate immune cells (e.g., DCs) via binding to either TLRs or CLRs. The activated DCs can direct cells of the adaptive immune system to facilitate promotion of Th2 cell response and subsequently increase risk of sensitization. Mannose receptor (MR), as a CLR, has been shown to mediate Bla g2 (purified cockroach allergen) uptake by DCs and to determine allergen-induced T cell polarization. Additionally, genetic factors may play an important role in conferring the susceptibility to cockroach sensitization. Several genes have been associated with cockroach sensitization and related phenotypes (*HLA-D, TSLP, IL-12A, MBL2*). In this review, we have focused on studies on the cockroach allergen induced immunologic responses and genetic basis for cockroach sensitization.

## 1. Introduction

**Figure 1:**
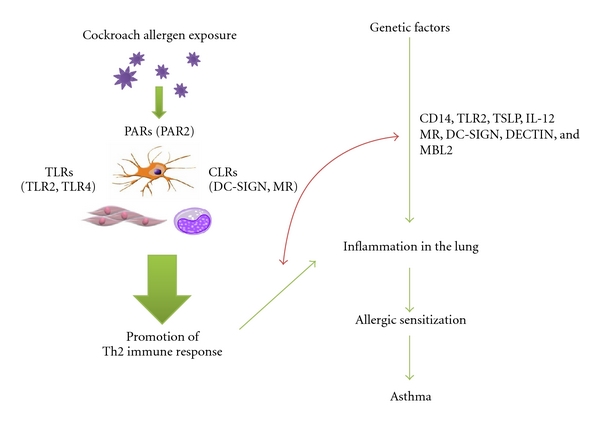
The mechanism of cockroach allergen-induced allergic sensitization. Cockroach-derived proteases can disturb airway epithelial integrity and lead to an increased penetration of cockroach allergen, which can activate innate immune cells (e.g., dendritic cells (DCs)) via binding to Toll-like receptors (TLRs) or C-type lectin receptors. The activated DCs can direct cells of the adaptive immune system to a promotion of Th2 cell response and subsequently increase risk of sensitization. On the other hand, genetic factors, particularly genetic variants in TLRs, CLRs, CD14, either alone or in interaction with cockroach exposure, confer the susceptibility to increased risk of cockroach sensitization and subsequently inflammation in the lung and asthma.

Asthma prevalence has markedly increased worldwide over the past three decades [1]. Exposure to indoor allergens is known to exacerbate asthma. Asthma symptoms due to exposure to cockroaches have been recognized since the 1940s. Scientific studies over the years have demonstrated that cockroach allergen is one of the major risk factors for the development of asthma [2–4]. Particularly, cockroach allergen exposure appears to have a greater effect on asthma morbidity than that of dust mite or pet allergen among inner-city children with asthma [5–7]. However, while there appears to be a rather clear relationship between allergen exposure and allergen sensitization or respiratory symptoms, the dose-response relationship is most relevant for “susceptible” individuals [7, 8]. Furthermore, a segment of the population, even when exposed to very high concentrations of allergen, will never become sensitized [9]. These studies suggested that there may be a genetic basis for allergen sensitization which contributes to the risk of asthma and/or the severity of asthma. It was recognized that interaction between gene and environment may control the development of asthma, but little is known regarding the causal relationship between cockroach exposure, sensitization, and asthma. A possible mechanism for the cockroach allergen induced allergic sensitization is illustrated in Figure 1. Cockroach allergen contains and produces many proteins and macromolecules, such as proteases [10, 11]. Cockroach-derived protease can disturb airway epithelial integrity and leads to an increased penetration of allergen proteins, resulting in activation of innate immune cells (e.g., dendritic cells (DCs)), which will direct cells of the adaptive immune system to Th2 cell development, lead to the lung inflammation and, subsequently, increased risk of sensitization [12, 13]. Protease-activated-receptor- (PAR-) 2, a receptor for protease, has been shown to mediate activation of airway epithelial cells [14, 15], and development of allergic diseases [16, 17]. Studies on PAR-2 deficient mice have demonstrated that PAR-2 mediates allergen-derived proteases in cockroach frass-induced airway allergic inflammation [18]. On the other hand, proteases may also serve as ligands for pattern recognition receptor (PPR). It was evident that German cockroach frass contains a Toll-like-receptor- (TLR) 2 ligand because it directly affected neutrophil cytokine production via TLR-2 [19, 20]. Furthermore, C-type lectin receptors (CLRs) are crucial in recognition of complex glycan structures and facilitate the endocytosis and presentation of pathogens [21–23]. Mannose receptor (MR), as a CLR, has been shown to mediate the uptake of diverse native allergens by DCs and to determine allergen-induced T-cell polarization [24, 25]. Significant binding of allergens and allergen extracts with variable binding activities to DC-SIGN and its receptor, L-SIGN, have been recently demonstrated [26]. Our recent studies have explored the mechanisms for cockroach allergen-induced allergic sensitization, including investigation of the Th1/Th2 cytokine profile of cocultured plasmacytoid dendritic cells (pDCs) and CD4^+^ T-cells and identification of the “transcript signatures” for the immune response to cockroach allergen using high-throughput expression profiling of cocultured cells [27]. Furthermore, we performed initial genome-wide association studies (GWASs) for cockroach sensitization among African Americans. This paper focuses on studies on the cockroach allergen-induced immune response and genetic basis for cockroach sensitization.

## 2. Cockroach Allergen Exposure and Sensitization and Risk of Asthma

Indoor allergens associated with the development of asthma include those derived from cockroach [28], house-dust mites [29], animal dander [30], and mold spores [31]. Among them, cockroach allergen exposure is a strong risk factor for asthma associated with increased frequency and severity of childhood allergies and asthma among inner-city children [5, 6, 32]. For example, in the children's bedrooms, 50.2% had cockroach allergen levels that exceeded the disease-induction threshold, compared with 9.7% for dust mite allergen levels and 12.6% for cat allergen levels. The rate of hospitalization for asthma was 3.4 times higher among children who were skin test positive to cockroach antigen and whose bedrooms had high levels of cockroach allergen. The same group also had 78% more visits to health care providers, experienced significantly more wheezing, and missed more school because of asthma compared to the children who were skin test negative to cockroach allergen. Early life exposure to cockroach allergen can lead to allergic sensitization [1, 32], which also has been associated with an increased risk for persistent asthma and bronchial hyperresponsiveness and with a greater loss of function [33, 34]. Studies from the Inner-City Asthma Consortium showed that allergen-specific IgE levels were correlated with allergen exposure among sensitized participants (*P* < 0.0001 for cockroach), and specific IgE levels for cockroach are also correlated with a range of inflammatory, physiologic, and clinical markers, suggesting that the allergen-specific IgE level could be a surrogate measure of the combination of sensitization plus degree of exposure, and ultimately asthma severity [35]. Similarly, in the New York City Neighborhood Asthma and Allergy Study (NAAS), Chew et al. found that increased allergen exposure was associated with increased probability of sensitization (IgE) to cockroach (*P* < 0.001) [36], and cockroach allergen (Bla g2) was more prevalent in the bed dust taken from the homes in the high asthma prevalence neighborhoods (HAPNs) compared with low asthma prevalence neighborhoods (LAPN), while sensitivity to cockroach allergen was twice as common at 23% versus 10% [7]. These studies further supported the notion that cockroach allergen exposure increases the risk of allergic sensitization, which is in turn related to the development of asthma. Importantly, it is worthwhile to note that the combination of cockroach sensitization and exposure to high levels of this allergen increased the frequency of asthma-related health problems overall in the inner city environment when compared with either of them alone, suggesting that allergic sensitization is a specific, major contributor to asthma morbidity for individuals with high exposure [5, 6].

## 3. Cockroach Allergen and Protease-Activated Receptors (PARs)

Environmental factors, including cockroach, house dust mite, and mouse, are thought to be risk factors for asthma. In particular, exposure to high levels of cockroach allergens in the home is a major risk factor for symptoms in sensitized individuals. Cockroach allergen is believed to derive from feces, saliva, and the bodies of these insects. Both *Blatella germanica* (German cockroach) and *Periplaneta Americana *(American cockroach) are important producers of major cockroach allergens [37]. German cockroach is especially ubiquitous, particularly in large, crowded cities in the United States [38]. However, it remains unclear how the cockroach allergens induce allergic sensitization and asthma. Cockroach allergen, like many of other allergens, HDM, fungi, pollen, and cat, contain and produce many proteins and macromolecules, such as proteases. Indeed, protease activities were detected in German cockroach frass and whole-body extract [10, 11]. It was suggested that cockroach-derived proteolytic enzymes disturb airway epithelial integrity, resulting in increased penetration of allergen proteins and increased risk of sensitization [12, 13]. Proteases may serve as ligands for PARs that mediate activation of airway epithelial cells and lead to the release of TNF, IL-8, and IL-6 [14, 15]. PAR-2, a major member in a family of proteolytically activated G-coupled receptors, has been associated with allergic diseases [16, 17]. Recent studies found that proteases from *A. alternata* act through PAR-2 to induce rapid increases in human airway epithelial [Ca2+]i *in vitro *and cell recruitment *in vivo, suggesting* critical early steps in the development of allergic asthma [39]. In addition, activation of PAR-2 was shown to increase the expression of thymic stromal lymphopoietin (TSLP), which activates DCs to polarize naive T-cells to Th2 cells [40]. Further studies on PAR-2 deficient mice have demonstrated that PAR-2 mediates allergen-derived proteases in cockroach frass-induced airway allergic inflammation, including increased airway hyperresponsiveness, Th2/Th17 cytokine release, serum IgE levels, cellular infiltration, and mucin production, but the effect was only observed when allergen was administered through the mucosa [18]. Collectively, these data suggest that proteases may link the innate and adaptive immune responses via PAR-2. In contrast, proteases may also serve as ligands for pattern recognition receptor (PPR). It was evident that German cockroach frass contains a TLR2 ligand, which actives neutrophils [19] and leads to release of MMP-9 and decreased allergic responses to cockroach frass [20]. However, it still remains uncertain about the presence and activities of proteases in cockroach extract, because neither serine protease inhibitor nor cysteine protease inhibitor can inhibit PAR-2 cleavage by cockroach extracts [41]. This was consistent with the studies on one of the purified cockroach allergens, Bla g2. Bla g2 has been shown to be a major antigen according to the investigation of IgE-mediated response (60%). Although Bla g2 shares sequence homology with the aspartic proteinase family of proteolytic enzymes, it lacks proteolytic activity in a standard milk-clotting assay using casein as a substrate [42].These findings suggest that it may be enzymatically inactive factors, other than enzymatic activity, which play a role in cockroach-induced immunological response.

## 4. The Immunological Role of Dendritic Cells (DCs) in Shaping the Immune Response

**Figure 2:**
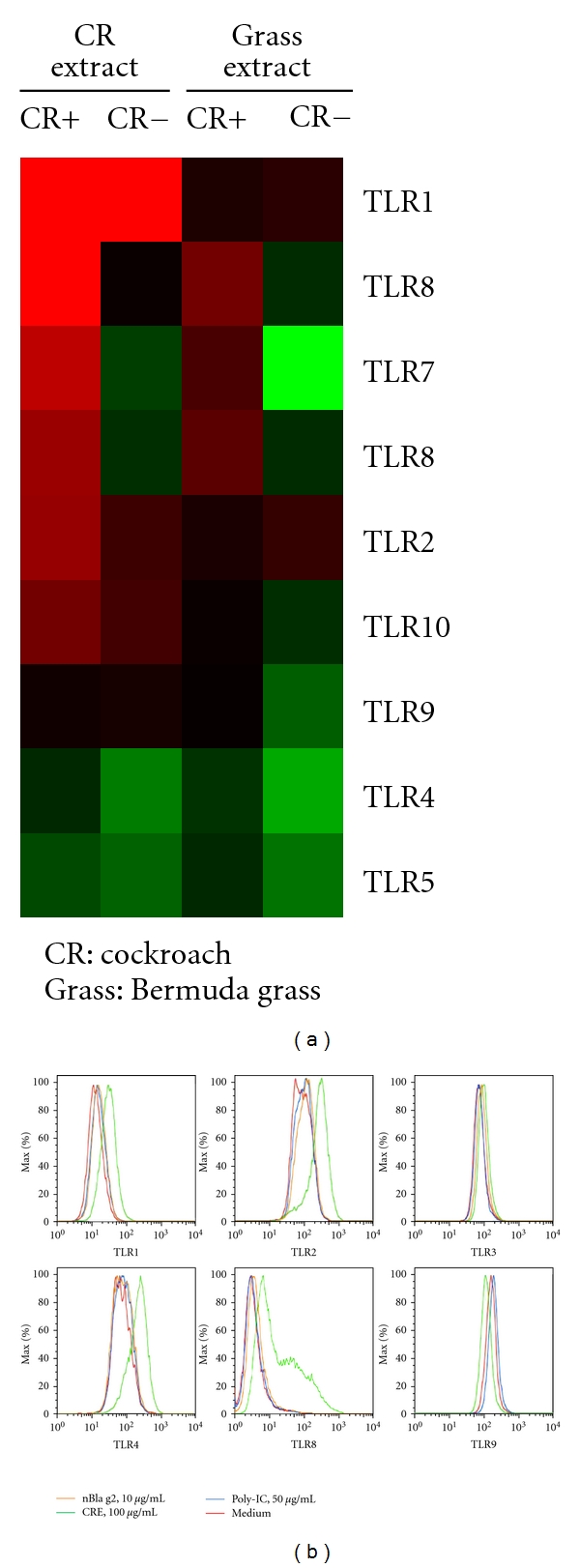
*TLR *gene expression in cocultured pDCs and CD4^+^ T cells and human THP-1 cells. (a) Microarray analysis of TLR transcripts expressed in cockroach allergen (CR) and Bermuda grass allergens treated cocultured pDCs and CD4^+^ T cells from cockroach-sensitized and -nonsensitized subjects. Upregulated genes are represented in red and downregulated genes in green. (b) TLR expression in THP-1 cells was detected at the protein levels by FACS (red, medium; orange: nBla g2; green: CRE, 100 ug/mL; blue: Poly-IC, 50 ug/mL).

DCs are the most powerful antigen-presenting cells (APCs) that process cockroach antigen and play a critical role in the initiation of the immune response and T-cell polarization [43–45]. Animal models have suggested that DCs are vital for both initiation and maintenance of allergic airway inflammation in asthma [46]. There are two major subsets of immature DCs that circulate in blood, namely, the CD11c^+^, CD123^low^ myeloid DCs (mDCs), and CD11c^−^, CD123^high^ plasmacytoid DC (pDCs). There is accumulating evidence from animal models that mDCs have a crucial role in the development of allergic asthma [47, 48]. In particular, Mo et al. found an increased airway hyperresponsiveness, eosinophil counts, and Th2 cytokines in BAL after intratracheal administration of OVA-pulsed mDCs [49]. In contrast, pDCs have been reported to inhibit allergic airway inflammation and Th2-type cytokine production in a mouse model of asthma [19], or play a limited role in priming T-cells in the mouse model of asthma [49]. It seemed that the interaction between pDCs and mDCs might control Th1/Th2 balance with a proallergic role for mDCs and antiallergic properties of pDCs. However, human pDCs can also stimulate allergen-dependent T-cell proliferation and Th2-type cytokine production as efficiently as mDCs [50]. In patients with atopic rhinitis, dermatitis, and asthma, there is a strong local increase in pDCs after allergen challenge [51–54]. It is possible that both pDCs and mDCs triggering either Th1-type or a Th2-type immune response may depend on the local microenvironment and stimulus. This was supported by our recent studies demonstrating that cocultured pDCs and CD4^+^ T cells produce significantly elevated levels of IL-13, IL-10, and TNF-*α*, but undetectable levels of IL-12p70, upon exposure to cockroach extract [27]. Furthermore, the increased levels of IL-13 were found in cells from cockroach allergic subjects when compared with cockroach nonallergic individuals. To identify the major players in the DC-mediated initiation of the immune response and T-cell polarization allergic disease, we performed gene array analyses (24000 transcripts and variants) in cocultured pDCs and CD4^+^ T cells aimed at identifying the “transcript signature” responsible for the initiation of the immune response and T-cell polarization. We found more than 50 genes uniquely expressed in cockroach treated cells, including *CD14, S100A8, CCL8*, IRF7, and *IFI44L*. Among these, *CD14* is one of the most replicated genes associated with asthma and associated traits [55]. A functional polymorphism in the promoter of *CD14* has been shown to modulate specific responses to environmental aeroallergens, at least among individuals predisposed to atopy [56]. Most importantly, pathway analysis suggested that both IFN and TLR signaling pathways are two major pathways in cockroach allergen-induced immunological responses. It is well known that TLRs, transmembrane proteins, highly expressed in DCs, play an important role in mediating allergen-induced innate and adaptive immune response [53]. Exogenous antigen presentation by DCs in the absence of direct TLR stimulation generally leads to tolerance [57]. Moreover, efficient generation of effector T-cell responses by DCs is dependent on the presence of TLR ligands in the phagosome containing the antigen being presented [58], suggesting that TLR signaling is critical in mediating antigen-induced adaptive immune response. It is likely that cockroach allergens interact with DCs via TLRs and lead to DC maturation, cytokine production, and APC function in T-cell polarization. Among all the TLR genes in our initial gene array analysis, TLR2, TLR3, TLR7, and TLR8 were upregulated in the cockroach allergic group compared with cockroach nonallergic group (Figure 2(a)). Of these, increased TLR2 and TLR8 were also validated at the protein levels (Figure 2(b)), suggesting that TLR2 and TLR8 may be important TLRs for cockroach sensitization. Indeed, recent report has provided strong evidence that TLR2 and TLR8 may confer susceptibility to asthma and related atopic disorders [19, 59]. In particular, German cockroach contains a TLR2 agonist and directly activates cells of the innate immune system, which may be critical in linking innate and adaptive immunity [19]. Genetic variation in TLR2 (rs4696480) has been identified as a major determinant of the susceptibility to asthma and allergies in children of farmers.

**Figure 3:**
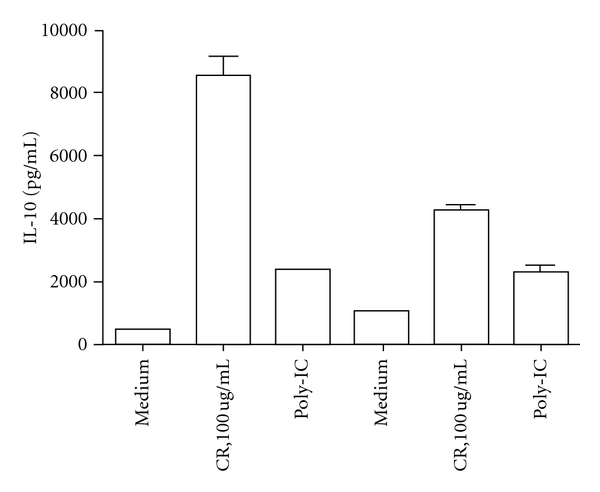
Cockroach allergen induced-IL-10 secretion in mDCs. IL-10 production was detected in the cockroach-extract- (CR-) treated alone (100 ug/mL) or together with anti-DC-SIGN mDCs. IL-10 levels were measured by ELISA.

C-type lectin receptors (CLRs), on the other hand, are crucial in recognition of complex glycan structures on various pathogens and have evolved to facilitate the endocytosis and presentation of pathogens [21–23]. In fact, signaling through CLRs has been shown to be able to induce T-cell activation and tolerance and modify the cellular response via cross-regulation of the TLR-mediated effect [23]. These regulatory functions have been clearly exemplified by three members of the CLRs, DC-SIGN (dendritic cell-specific, CD209), L-SIGN (CD299), and MR [60, 61]. Thus, distinct DC subsets with different sets of CLRs may recognize distinct classes of antigens to induce tolerance or activate immunity, wherein complex glycan structures on antigens may play a key role. While the direct interaction between allergens and CLRs has not been demonstrated, the mere fact that most allergens contain complex glycan structures raises the possibility that allergen-CLR signaling may modulate DCs and subsequent immune response. Indeed, MR has been shown to mediate the uptake of diverse native allergens by DCs and determines allergen-induced T-cell polarization through modulation of indoleamine 2,3 dioxygenase (IDO) activity [24]. In addition, Emara et al. showed that Fel d 1 interacts with immune cells by MR, and found that MR probably plays a pivotal role in allergic response to Fel d 1 [25]. Study on peanut allergens has provided a suggestive evidence that one of the major allergens, Ara h1, is able to polarize Th2 response via its likely interaction with DC-SIGN on monocyte-derived DCs [62]. We also found that mDCs produced a large amount of IL-10 after treatment with German cockroach extract, and that the increased expression was blocked by anti-DC-SIGN (Figure 3), suggesting that DC-SIGN in mDCs mediates cockroach allergen-induced allergic response. Hsu et al. demonstrated significant binding of allergens and allergen extracts with variable binding activities to DC-SIGN and its receptor, L-SIGN [26]. These allergens include bovine serum albumin (BSA) coupled with a common glyco-form of allergens and a panel of purified allergens (BG60 from Bermuda grass pollen, Der p2 from house dust mite). Interaction between BG60 and DC-SIGN-activated Raf-1 and ERK kinases and led to the induction of TNF-*α* expression. These studies identified an important signaling pathway for allergen-induced immunity, and, importantly, they suggested that there may be a cross-regulation between CLRs, TLRs, and PAR2.

## 5. Genetic Basis for Cockroach Sensitization

**Figure 4:**
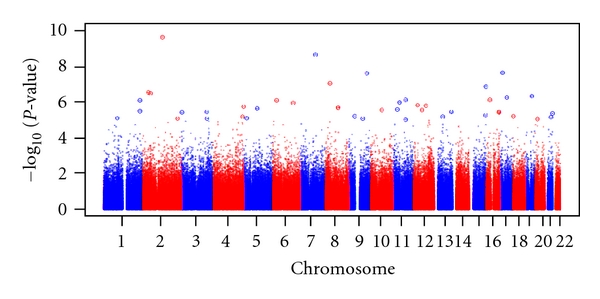
Overview of genome-wide association study of cockroach sensitization in the African American population. Manhattan plot showing the association of 644,709 SNPs by chromosome for cockroach allergy versus –log⁡_10_⁡*P* value. The *x*-axis represents genomic position, and the *y*-axis shows –log⁡_10_⁡(*P*).

While there appears to be a rather clear relationship between allergen exposure and allergen sensitization, the dose-response relationship is most relevant for “susceptible” individuals [1, 8]. Conversely, the majority of individuals, when exposed to very high concentrations of allergen, never become sensitized [9]. Indeed, one of our previous studies has implied a role for genetic susceptibility wherein cockroach sensitization was found to be more prevalent among African Americans compared with European Americans living in the Baltimore-Washington, DC, metropolitan area, even after controlling for socioeconomic status [63] These findings suggest that cockroach sensitization is not a function of cockroach allergen exposure alone, and that genetic susceptibility may be important. Indeed, significant familial aggregation of allergic sensitization to cockroach allergen has been observed in the Chinese population [64]. In a genome-wide linkage study of asthma-related phenotypes on 2,551 individuals from 533 families, Xu et al. provided suggestive evidence of linkage at D4S1647 for skin reactivity to cockroach defined by skin prick tests (SPTs) (pointwise *P* = 0.0003) [65]. Hunninghake et al. recently reported significant evidence of linkage to cockroach-specific IgE on chromosome 5q23 (peak LOD, 4.14 at 127 cM) [66]. Within this genomic region, there is a compelling candidate gene with experimental evidence of female-specific effects on lung disease, thymic stromal lymphopoietin (TSLP). In a sex-stratified analysis, the T allele of single-nucleotide polymorphism (SNP) rs2289276 in the 5'untranslated region of *TSLP* was associated with reductions in IgE concentrations to cockroach. Interestingly, the same *TSLP* SNP rs2289276 also showed significant association with lower levels of total IgE (tIgE,*P* = 6.24 × 10^−6^) in our initial analyses of GWAS for tigE among cockroach allergic individuals. In a study on *HLA-D* associations and cockroach sensitization, Donfack et al. [67] observed associations with alleles of the *HLA-DR* molecule, DRB1*0101 in Hutterites and DRB1*0102 in African Americans, and hypothesized that the DRB1*0102 allele may have a higher affinity for cockroach allergens and elicit a stronger response to bind antigens than DRB1*0101 allele. Leung et al. observed that polymorphisms in the *Mannose-binding lectin* (*MBL*) gene may protect against cockroach sensitization in Chinese children [68], and Pistiner et al. demonstrated that polymorphisms in *IL12A* were associated with cockroach sensitization among children with asthma in both Costa Rica and European-ancestry children with asthma in the Childhood Asthma Management Program (CAMP) [69]. We performed a genome-wide association analysis for cockroach sensitization in the African American population. A summary of the results is shown for a trend in the association between cockroach sensitization and each SNP measured in the GWAS (Figure 4). Overall, there were 7,768 SNPs in 4,018 genes with *P* value < 0.01. When specifically limiting the SNPs to those at *P* < 0.001, we found at least 12 genes that had differentially gene expression in our gene array analysis for cockroach allergen exposure (*IFI44, CTLA4, LYN, BCL6, CCL1, MERCK, HERC6, TRIB1, DNAPTP6, SAMSN1, RAFTLIN*, and *GMZB*). Among those, *CTLA4* [70], *BCL6* [71], *GZMB* [72], and *CCL1* [73] have been associated with allergy and asthma and related phenotypes. The results suggested that integrating GWAS with gene expression profiling studies will be useful approach to identify candidate for cockroach allergic sensitization. 

## 6. Conclusion

Asthma is a major public health concern. Cockroach allergen exposure and cockroach allergic sensitization could contribute to the higher prevalence of asthma. Although studies on the causal relationship between cockroach allergen exposure, sensitization, and asthma are very limited, several receptors (*PAR-2, TLRs, CLRs*) and their pathways have been seen to be important in mediating antigen uptake from the environment and inducing allergies by signaling T-cells to activate an inappropriate immune response. In particular, cockroach-derived protease can disturb airway epithelial integrity via PAR-2 and leads to an increased penetration of cockroach allergen, resulting in activation of innate immune cells (e.g., DCs) via binding to either *TLRs* or *CLRs*. The activated DCs can direct cells of the adaptive immune system to facilitate promotion of Th2 cell response and subsequently increase risk of sensitization. However, it remains largely unknown whether different cell types expressing different sets of receptors may recognize distinct classes of cockroach allergens to induce different immune responses, and whether these receptors have a cross-regulation. On the other hand, genetic factors, particularly genetic variants in *TSLP, MBL2, CD14,* and* IL-12A* have been associated with cockroach sensitization and related phenotypes. It would be of interest to study whether these genes in interaction with cockroach exposure confer an increased susceptibility to the risk of cockroach sensitization when compared with these genes analyzed alone. Continuous studies, we believe, on cockroach allergen-induced innate immunity and gene-environment interaction will add value to the existing research investment in these studies and offer novel insight into the molecular mechanisms that cause cockroach sensitization and subsequently asthma.

##  Funding 

This research was supported by NIH Grant 5R21A10884066.
