# Erratum to “Sensitization to Cockroach Allergen: Immune Regulation and Genetic Determinants”

**DOI:** 10.1155/2012/631847

**Published:** 2012-08-23

**Authors:** Peisong Gao

**Affiliations:** Department of Medicine, Johns Hopkins Asthma and Allergy Center, Johns Hopkins University School of Medicine, Baltimore, MD 21224, USA

In the paper entitled “Sensitization to cockroach allergen: immune regulation and genetic determinants,” there was an error in Figure 4 and this is the correct form. In addition the author wishes to acknowledge that data was partially supported by NIH grants AI50024 (Kathleen C. Barnes) and HL087699 (Kathleen C. Barnes).

## Figures and Tables

**Figure 4:**
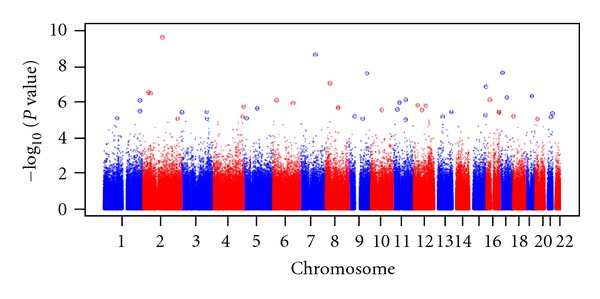
Overview of genomewide association study of cockroach sensitization in the African American population (Gao et al. JACI 2008; 121(2):S81). Manhattan plot showing the association by chromosome for cockroach sensitization versus −log⁡_10_⁡*P* value. The *x*-axis represents genomic position, and the *y*-axis shows −log⁡_10_⁡*P* value.

